# Connexin43 in mesenchymal lineage cells regulates body adiposity and energy metabolism in mice

**DOI:** 10.1172/jci.insight.170016

**Published:** 2024-02-13

**Authors:** Seung-Yon Lee, Francesca Fontana, Toshifumi Sugatani, Ignacio Portales Castillo, Giulia Leanza, Ariella Coler-Reilly, Roberto Civitelli

**Affiliations:** Department of Medicine, Division of Bone and Mineral Diseases, Musculoskeletal Research Center, Washington University School of Medicine, St. Louis, Missouri, USA.

**Keywords:** Metabolism, Adipose tissue, Adult stem cells, Obesity

## Abstract

Connexin43 (Cx43) is the most abundant gap junction protein present in the mesenchymal lineage. In mature adipocytes, Cx43 mediates white adipose tissue (WAT) beiging in response to cold exposure and maintains the mitochondrial integrity of brown adipose tissue (BAT). We found that genetic deletion of *Gja1* (Cx43 gene) in cells that give rise to chondro-osteogenic and adipogenic precursors driven by the *Dermo1*/*Twist2* promoter led to lower body adiposity and partial protection against the weight gain and metabolic syndrome induced by a high-fat diet (HFD) in both sexes. These protective effects were related to increased locomotion, fuel utilization, energy expenditure, nonshivering thermogenesis, and better glucose tolerance in conditionally *Gja1*-ablated mice. Accordingly, *Gja1*-mutant mice exhibited reduced adipocyte hypertrophy, partially preserved insulin sensitivity, increased BAT lipolysis, and decreased whitening under HFD. This metabolic phenotype was not reproduced with more restricted *Gja1* ablation in differentiated adipocytes, suggesting that Cx43 in adipocyte progenitors or other targeted cells restrains energy expenditures and promotes fat accumulation. These results reveal what we believe is a hitherto unknown action of Cx43 in adiposity, and offer a promising new pharmacologic target for improving metabolic balance in diabetes and obesity.

## Abstract 2

Adipocytes are coupled by gap junctions formed by connexin43, and lack of connexin43 in mesenchymal lineage cells is protective against the metabolic stress produced by a high-calorie diet.

## Graphical abstract

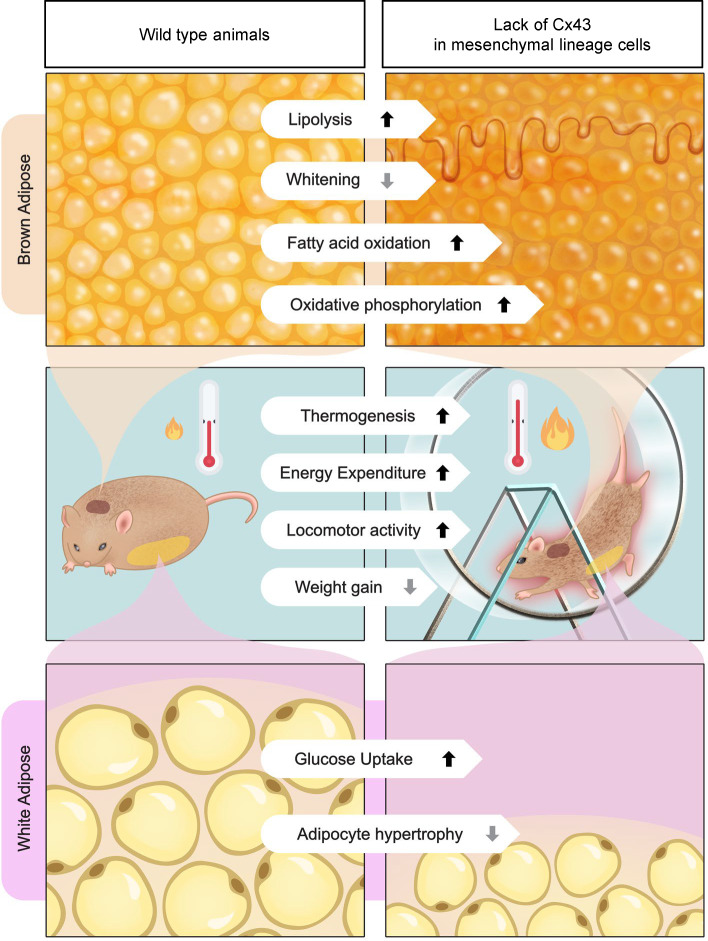

## Introduction

Adipose tissue is a metabolically active organ involved in many aspects of normal physiology, including tissue nutrient homeostasis and energy metabolism (1). The size of an individual’s adipose tissues depends on developmental and environmental factors that affect the number and size of adipocytes. Indeed, body adiposity can increase by either volumetric expansion of existing adipocytes (hypertrophy) or an increased number of adipocytes (hyperplasia). The balance between hypertrophic expansion of existing adipocytes and adipogenic differentiation is an integral component of metabolism and affects an individual’s health (2).

White adipocytes make up the bulk of fatty tissue in most animals, their main role being long-term energy storage in fat depots, which are referred to as white adipose tissue (WAT). In conditions of increased energy needs, white adipocytes can activate lipolysis and release free fatty acids from triglycerides. WAT also has an endocrine function, producing leptin, adiponectin, and other adipokines that regulate fuel utilization in peripheral tissues and lipid metabolism (3). Brown and beige adipocytes are instead primarily involved in energy consumption and heat dissipation. Brown adipocytes are found in a specific depot, the brown adipose tissue (BAT), while beige adipocytes are present in WAT under conditions of increased energy demands. The BAT contributes to lowering circulating lipids by favoring triglyceride uptake via lipoprotein lipase (4), fatty acid uptake from triglyceride-rich lipoproteins, and enhanced hepatic clearance of cholesterol-enriched molecules (5). Thus, the adipose tissue functions to balance an individual’s needs for energy storage, utilization, and dissipation as heat.

Our work explores the role of a gap junction protein in adipose tissue function. Gap junctions are intercellular channels that allow aqueous continuity between connected cells. They are formed by juxtaposition of 2 transmembrane hemichannels in apposing cells, with each hemichannel composed of a homo- or heterohexamer of connexin proteins. Gap junctions have been observed in adipose tissue during development (6, 7) and BAT adipocytes are more functionally connected than WAT adipocytes in adult mice (8). However, the specific function of gap junctions in the different adipose tissues remains to be fully elucidated. Among the 21 connexins present in humans (9), connexin43 (Cx43) is the main gap junction protein expressed in mesenchymal lineage cells (9, 10). Work using adipocyte-targeted *Gja1* (Cx43 gene) ablation in mice has shown that Cx43 mediates adipocyte beiging in response to sympathetic neuronal signals, and that overexpression of Cx43 promotes WAT beiging even with mild cold stimuli (11). Moreover, *Gja1* deletion in mature adipocytes disrupts mitochondrial integrity via autophagy, resulting in metabolic dysfunction in mice kept on a high-fat diet (HFD) (12). These results suggest that Cx43 in mature adipocytes has beneficial roles in controlling energy metabolism and thermogenesis.

In our studies on Cx43 in the skeleton, we had observed that mice with ablation of *Gja1* broadly in mesenchymal lineage cells, using the *Dermo1*/*Twist2* promoter, acquire less body weight with age, in addition to having undermineralized, thinner long bones relative to control mice (13). Using these genetically engineered mice, here we show that, contrary to expectations, lack of Cx43 in mesenchymal lineage cells is protective against the metabolic stress produced by a high-calorie diet, whereas more restricted *Gja1* ablation in mature adipocytes is not. Specifically, we demonstrate that lack of Cx43 reduces white adipocyte hypertrophy, increases glucose uptake and insulin sensitivity, prevents BAT whitening, and increases locomotor activity, energy expenditure, nonshivering thermogenesis, and BAT lipolysis. Thus, Cx43 has complex and cell-specific functions in adipose tissue biology and energy metabolism.

## Results

### Gja1 ablation in mesenchymal lineage cells results in metabolic benefits in mice on a standard diet.

To study the role of Cx43 in mesenchymal progenitors, we used *Dermo1/Twist2-Cre:Gja1^fl/fl^* (cKO^Tw2^) mice, which we have previously described (13, 14). *Dermo1/Twist2-Cre* targets the entire skeleton, muscle, and epidermis at birth (Supplemental Figure 1A; supplemental material available online with this article; https://doi.org/10.1172/jci.insight.170016DS1). In adult *Dermo1/Twist2-Cre;Ai9* reporter mice, TdT signal was detected diffusely in BAT and WAT adipocytes, including cells in the stromal/perivascular areas, although vessels were not targeted (Supplemental Figure 1, B and C). A small proportion of hepatocytes were also targeted by *Dermo1/Twist2-Cre* (Supplemental Figure 1D), and in the spleen, TdT fluorescence was abundant in the stromal component, while the nodular areas were negative (Supplemental Figure 1E). Abundant X-gal staining of fat depots and in histologic sections of BAT and WAT confirmed effective *Dermo1/Twist2-Cre*–driven *Gja1* gene recombination in both fat tissues (Supplemental Figure 1, F–I). The more intense stain in BAT (Supplemental Figure 1, F′ and G′) most likely reflects higher cell density than in WAT (Supplemental Figure 1, H′ and I′).

**Figure 1:**
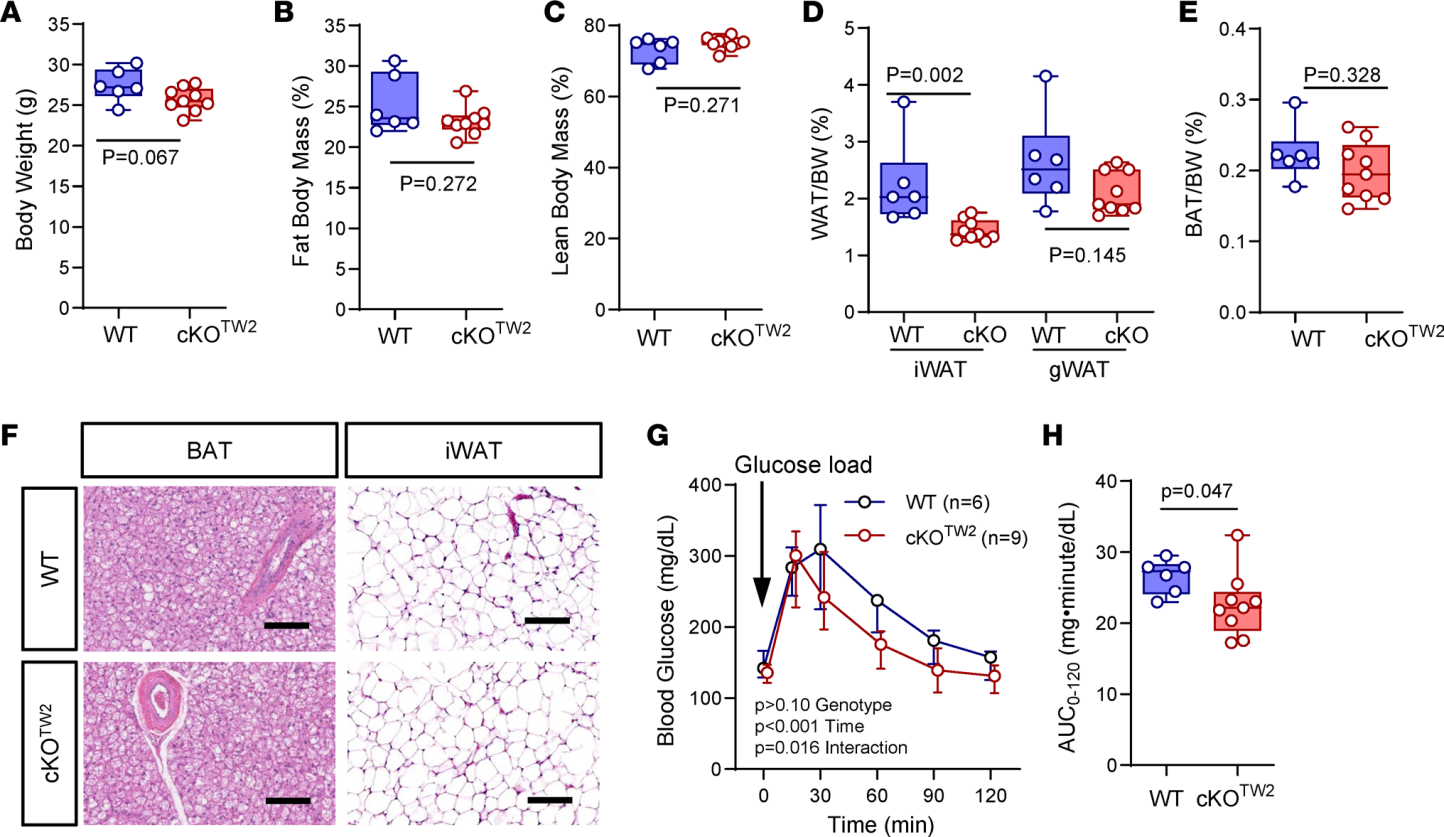
*Gja1* ablation in mesenchymal lineage cells leads to metabolic benefits in male mice on a standard diet. (**A**) Body weight and (**B**) dual-energy x-ray absorptiometry–determined (DXA-determined) percentage body fat and (**C**) lean mass in 7-month-old WT (blue) and cKO^TW2^ mice (red). (**D**) WAT weight normalized to body weight (BW) in inguinal (iWAT) and gonadal (gWAT) depots. (**E**) Weight of suprascapular BAT depots normalized to BW. (**F**) H&E-stained histologic sections of BAT and iWAT from WT and cKO^TW2^ mice. Scale bars: 100 μm. Each image is representative of 6 WT and 9 cKO^TW2^ mice. (**G**) Intraperitoneal glucose tolerance test: blood glucose before and after an intraperitoneal load of 1.5 g/kg D-glucose. (**H**) Areas under the curve (AUCs) calculated between 0 and 120 minutes for animals included in **G**. Data are presented as box-and-whisker plots representing the interquartile range (box bounds) with median (inside bar); whiskers represent maximum and minimum values. Groups in **A**–**E** and **H** were compared using 2-tailed Mann-Whitney *U* test. *P* values in **G** represent the effect of genotype, time, and their interaction by 2-way ANOVA (genotype, *F* = 3.082, *P* = 0.103; time: *F* = 97.14, *P* < 0.001; time × genotype: *F* = 3.032, *P* = 0.016).

At age 7 months, body weight was marginally lower in cKO^Tw2^ than in *Gja1^fl/fl^* (wild-type equivalent: WT) male mice (Figure 1A), and the difference was more pronounced among female mice (Supplemental Figure 2A). Neither fat mass nor lean mass was altered in mutant mice when assessed by dual-energy x-ray absorptiometry (DXA) (Figure 1, B and C, and Supplemental Figure 2, B and C). At necropsy, gonadal fat depots (Figure 1D and Supplemental Figure 2D) and BAT mass were similar in control and mutant mice (Figure 1E and Supplemental Figure 2F). However, the weight of inguinal fat depots from 7-month-old cKO^Tw2^ mice was lower than those excised from WT mice of both sexes (Figure 1D and Supplemental Figure 2E). Histologically, we observed no abnormalities in morphology or size of adipocytes in inguinal WAT (iWAT), or in BAT in mutant male mice (Figure 1F). Following an intraperitoneal (i.p.) glucose tolerance test (iGTT), male cKO^Tw2^ mice showed faster return to baseline of blood glucose compared with WT mice (Figure 1, G and H). Such differences were not seen in female cKO^Tw2^ mice, which instead were hyperglycemic after glucose load (Supplemental Figure 2, G and H). Indirect calorimetry showed no significant differences between genotypes in food consumption (Supplemental Figure 3, A and B, and Supplemental Table 1), energy expenditure (Supplemental Figure 3, C and D, and Supplemental Table 1), and respiratory exchange ratio (Supplemental Figure 3, E and F, and Supplemental Table 1). However, cKO^Tw2^ mice were more active than WT mice during dark hours (Supplemental Figure 3, G and H, and Supplemental Table 1).

### Cx43 is upregulated by HFD in BAT, while it is downregulated during white adipocyte differentiation.

**Figure 2:**
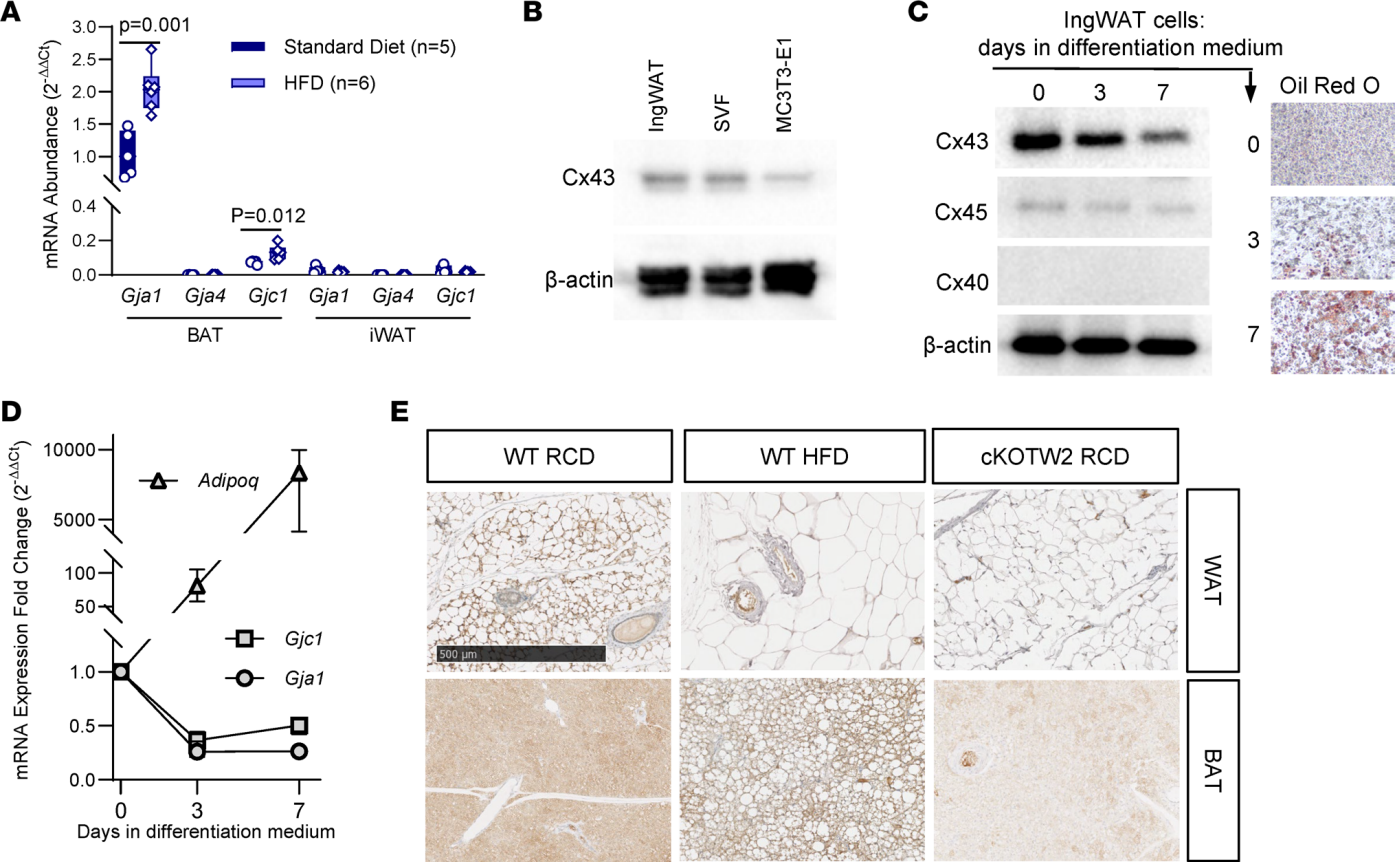
Cx43 is upregulated by HFD in BAT, and downregulated during adipogenic differentiation. (**A**) Expression of *Gja1* (Cx43), *Gja4* (Cx40), and *Gjc1* (Cx45) mRNA by qPCR in inguinal WAT (iWAT) and BAT in 5-month-old male mice fed either a regular chow diet (RCD) or HFD for 12 weeks. Data are presented as box-and-whisker plots representing the interquartile range (box bounds) with median (inside bar); whiskers represent maximum and minimum values. Groups were compared using 2-tailed Mann-Whitney *U* test. (**B**) Western blot of whole-cell lysates of confluent, undifferentiated cultures of IngWAT preadipocytic cells, cells isolated from the stromal vascular fraction (SVF) of WAT, and the osteogenic cell line, MC3T3-E1, as control. (**C**) Western blot of whole-cell lysates, Oil Red O stain, and (**D**) RT-qPCR analysis of mRNA of IngWAT cells before and during adipogenic differentiation (median and range, *n* = 6; *P* < 0.001 vs. time 0 at both time points). (**E**) Immunohistochemical staining (brown color) for Cx43 in WAT and BAT isolated from WT mice kept on either RCD or HFD and in cKO^TW2^ mice. Scale bar: 500 μm. Each image is representative of 3 mice per condition.

Cx43 is the most abundant connexin in the adipose tissue (8); it is upregulated during WAT beiging in mice (11) and during adipogenesis in human induced pluripotent stem cells (15). Using real-time quantitative PCR (RT-qPCR), we confirmed expression of *Gja1* in BAT and upregulation in mice fed an HFD, with fat providing 60% of calorie intake (Figure 2A). Of the other 2 connexins present in osteochondroprogenitor cells, *Gjc1* was also expressed in BAT and upregulated by HFD, even though it was approximately 10-fold less abundant than *Gja1* mRNA, whereas *Gja4* was not detected (Figure 2A). Although mRNA for *Gja1* and *Gjc1* was barely detectable in WAT extracts regardless of the diet (Figure 2A), Cx43 protein was abundantly expressed in preadipocytes obtained from the stromal vascular fraction (SVF) of WAT and in the IngWAT preadipocyte cell line (16) (Figure 2B). Consistent with the mRNA results, Cx45 was also present in preadipocytes, while Cx40 was undetectable; notably, both Cx43 and Cx45 protein and mRNA were rapidly downregulated upon in vitro adipocyte differentiation (Figure 2, C and D). Thus, low expression of Cx43 in differentiated adipocytes may explain the barely detectable *Gja1* mRNA in WAT extracts. Immunohistochemistry confirmed more intense Cx43-specific signal in BAT of HFD mice relative to mice kept on regular diet, and only background signal in BAT of cKO^Tw2^ mice (Figure 2E). Of note, Cx43-specific stain was absent in blood vessels, while it was visible in perivascular cells. Similarly, Cx43-specific stain was detected in WAT adipocytes kept on regular chow diet, without a notable increase in mice on HFD, whereas no Cx43-specific staining was detected in WAT of cKO^Tw2^ mice (Figure 2E).

### Gja1 ablation in mesenchymal lineage cells is partially protective against HFD-induced metabolic syndrome and fat accumulation.

**Figure 3:**
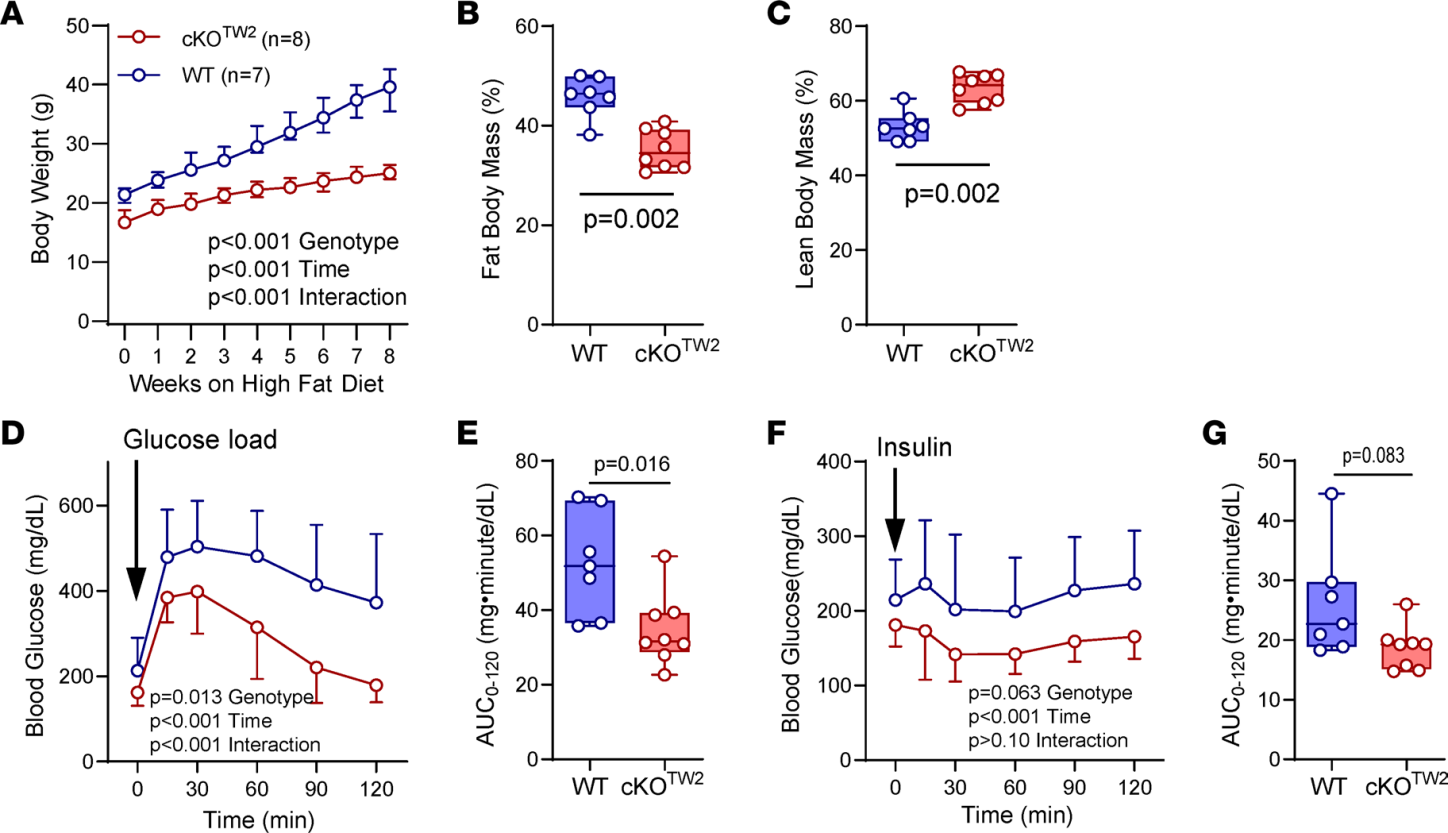
*Gja1* ablation in mesenchymal lineage cells is partially protective against HFD-induced obesity, hyperglycemia, and reduced glucose tolerance in male mice. (**A**) Body weight of 5-month-old WT (blue) and cKO^TW2^ (red) male mice during 8 weeks on HFD feeding. Data are shown as median and interquartile range; *P* values represent the effect of genotype, time, and their interaction by repeated-measures 2-way ANOVA (genotype, *F* = 53.53, *P* < 0.001; time: *F* = 288.3, *P* < 0.001; time × genotype: *F* = 42.08, *P* < 0.001). (**B**) Percentage body fat and (**C**) lean mass by DXA after HFD in the 2 genotype groups. (**D**) Intraperitoneal glucose tolerance test: blood glucose before and after an intraperitoneal load of 1.5 g/kg D-glucose (mean ± 95% CI; 2-way ANOVA: genotype, *F* = 8.202, *P* = 0.013; time: *F* = 70.87, *P* < 0.001; time × genotype: *F* = 5.972, *P* < 0.001). (**E**) Areas under the curve (AUCs) calculated between 0 and 120 minutes for animals included in **D**. (**F**) Intraperitoneal insulin tolerance test: blood glucose before and after an intraperitoneal injection of 0.75 U/kg insulin (mean ± 95% CI; 2-way ANOVA: genotype, *F* = 4.131, *P* = 0.063; time: *F* = 6.634, *P* < 0.001; time × genotype: *F* = 1.347, *P* > 0.10). (**G**) AUCs calculated between 0 and 120 minutes for animals included in **F**. Group data are in box-and-whisker plots representing the interquartile range (box bounds) with median (inside bar); whiskers represent maximum and minimum values. *P* values were determined by 2-sided Mann-Whitney *U* test.

We then investigated the consequences of Cx43 deletion on the metabolic response to obesogenic diets. Male mice fed an HFD for 12 weeks almost doubled their body weight, while cKO^Tw2^ mice experienced a far lesser weight gain on the same HFD (*P* < 0.001 for effect of diet by 2-way ANOVA; Figure 3A). Moreover, percentage body fat was significantly lower, and percentage lean weight was significantly higher in cKO^Tw2^ compared with WT male mice after HFD (Figure 3, B and C). Both WT and cKO^Tw2^ mice were hyperglycemic after 12 weeks on HFD. However, iGTT revealed a more efficient response in cKO^Tw2^ relative to WT mice, which remained severely hyperglycemic even 120 minutes after the glucose load (Figure 3, D and E). When HFD fed mice were injected with insulin i.p. (insulin tolerance test, iITT), blood glucose was significantly lower at all time points in cKO^Tw2^ mice compared with WT (Figure 3, F and G). Similarly, female cKO^Tw2^ mutants gained far less weight on HFD (Supplemental Figure 4A) and accumulated significantly less body fat (Supplemental Figure 4B), resulting in smaller peripheral fat depots (Supplemental Figure 4, C and D). The iGTT showed prolonged hyperglycemia in WT females fed HFD, while cKO^Tw2^ had significantly lower blood glucose levels (Supplemental Figure 4, E and F), thus reversing the trend seen in female mice kept on regular chow diet (see above); however, both mutant and WT females responded equally to an insulin bolus (Supplemental Figure 4, G and H). These results are also consistent with previous evidence that female mice are partially protected from the metabolic effects of HFD relative to male mice (17).

**Figure 4:**
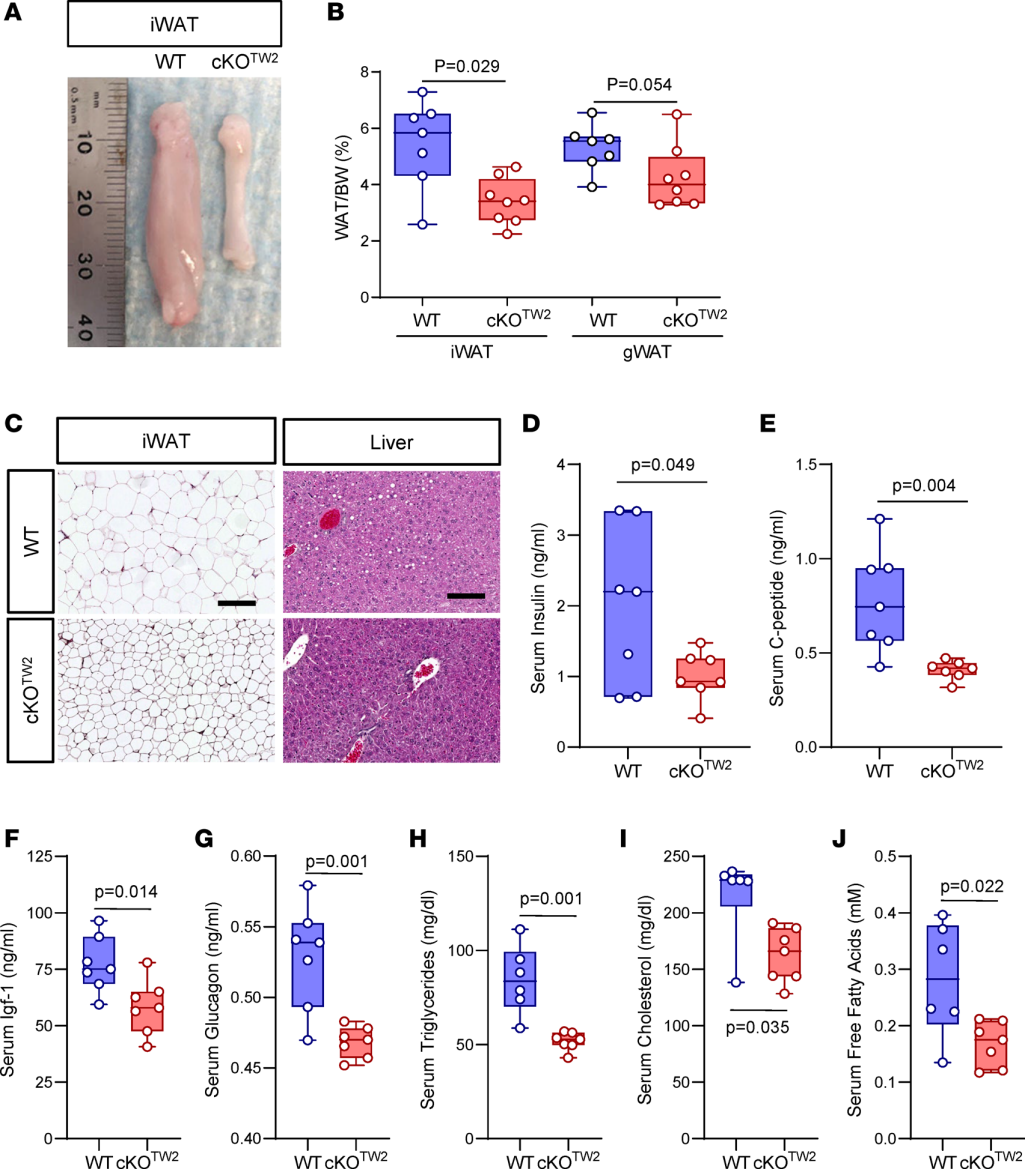
*Gja1* ablation in mesenchymal lineage cells protects HFD-induced expansion of fat depots and adipocyte hypertrophy in male mice. (**A**) Representative morphology of inguinal WAT (iWAT) in 2-month-old WT and cKO^TW2^ male mice after 12 weeks on an HFD. (**B**) Percentage iWAT and gonadal WAT (gWAT) relative to body weight in the 2 genotypes after HFD. (**C**) Representative H&E-stained histological sections of iWAT and liver of WT (*n* = 7) and cKO^TW2^ (*n* = 8) mice after 12 weeks on HFD. Scale bars: 50 μm. Serum levels of (**D**) insulin, (**E**) C-peptide, (**F**) Igf-1, (**G**) glucagon, (**H**) triglyceride, (**I**) cholesterol, and (**J**) fatty acid measured in WT and cKO^TW2^ mice after 12 weeks on HFD. Box-and-whisker plots represent the interquartile range (box bounds) with median (inside bar); whiskers represent maximum and minimum values. *P* values were determined by 2-sided Mann-Whitney *U* test.

Given the more severe metabolic phenotype, the following studies were performed only in male mice. Consistent with the reduced obesity, WAT mass was macroscopically smaller in cKO^Tw2^ relative to WT male mice after 12 weeks on HFD (Figure 4A). Expressed as percentage body weight, both inguinal and gonadal WAT (gWAT) depots were comparatively smaller in cKO^Tw2^ than in WT mice (Figure 4B). Importantly, H&E staining of iWAT sections revealed smaller sized adipocytes in cKO^Tw2^ mice, compared with larger cells in WT iWAT (Figure 4C). As histologic sections of liver showed mild steatosis in WT mice but no fat accumulation in cKO^Tw2^ mutants following HFD (Figure 4C), we can exclude a shift in fat accumulation into other organs in Cx43-deficient mice, as it occurs in lipodystrophy. Finally, serum insulin, C-peptide, Igf-1, and glucagon were all significantly lower in cKO^Tw2^ than in WT male mice (Figure 4, D–G), as were serum triglycerides, cholesterol, and free fatty acids (Figure 4, H–J). Overall, these results suggest that absence of Cx43 in the mesenchymal lineage is partially protective of the obesogenic effect and related metabolic abnormalities of high caloric dietary intake, a conclusion opposite to what has been reported when *Gja1* was ablated specifically in mature adipocytes (12).

### Gja1 ablation in mesenchymal lineage cells favors adipogenesis and glucose uptake in WAT of mice fed an HFD.

**Figure 5:**
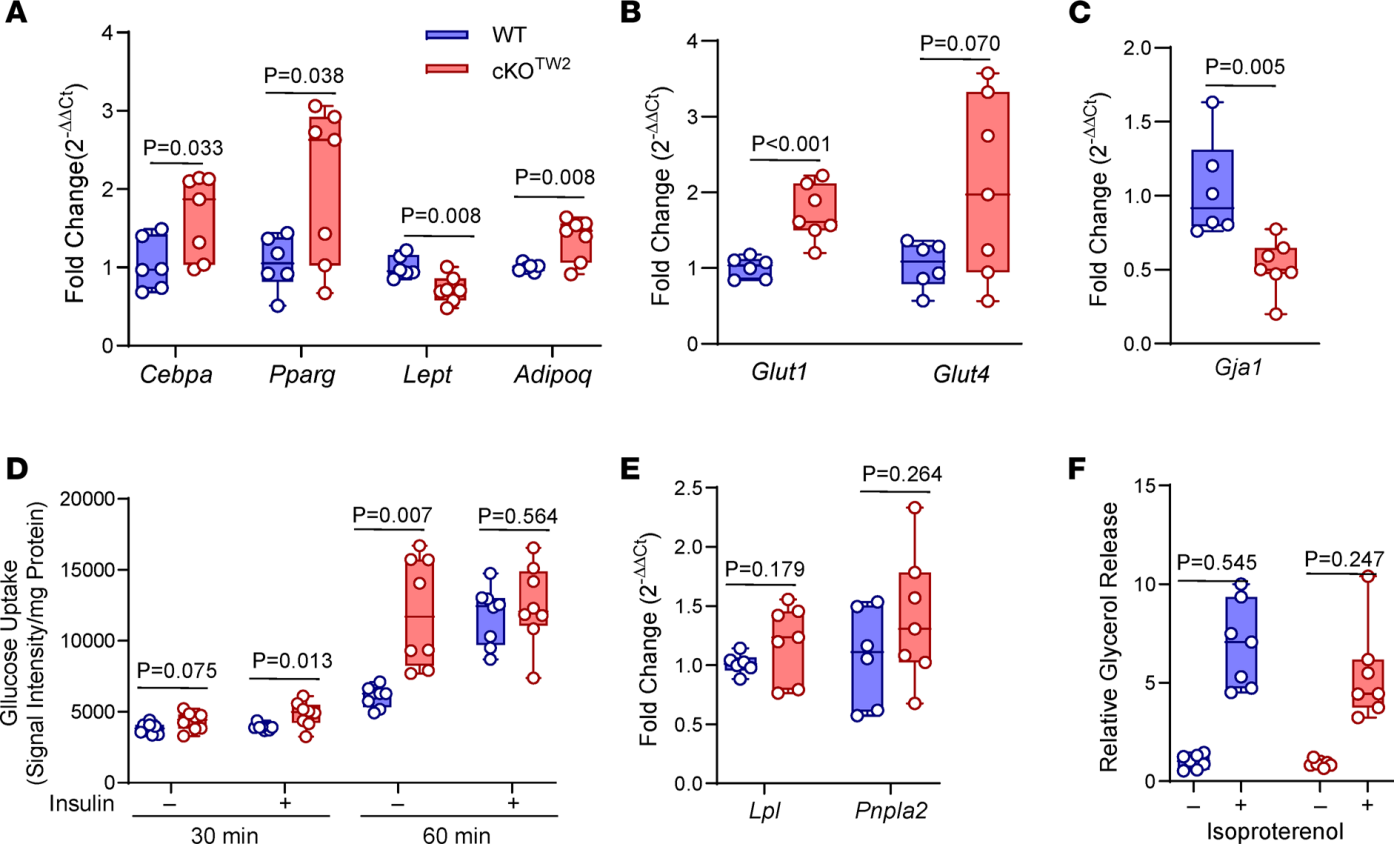
*Gja1* ablation in mesenchymal lineage cells upregulates adipogenic genes and promotes glucose uptake without altering lipolysis in WAT. (**A**) mRNA expression by RT-qPCR of adipogenic genes, (**B**) glucose transporters, (**C**) *Gja1*, (**D**) 2-NBD-glucose uptake, (**E**) lipolysis-associated genes, and (**F**) glycerol release relative to tissue weight, in the presence or absence of 10 μM isoproterenol in inguinal WAT (iWAT) of 5-month-old WT (blue) and cKO^TW2^ male mice (red) after 12 weeks on HFD. Box-and-whisker plots represent the interquartile range (box bounds) with median (inside bar); whiskers represent maximum and minimum values. *P* values were determined by 2-sided Mann-Whitney *U* test.

To study the mechanisms of the partial resistance of Cx43-deficient mice to the obesogenic HFD, we examined the expression of genes associated with adipogenesis, glucose uptake, and lipolysis. After HFD, *Cebpa*, *Pparg*, and *Adipoq* were higher in subcutaneous fat (iWAT) obtained from cKO^Tw2^ relative to control mice, whereas *Lep* was lower (Figure 5A). Expression of the 2 main glucose transporters, *Glut1* and *Glut4*, was also increased in iWAT of cKO^Tw2^ relative to WT mice (Figure 5B), while *Gja1* mRNA was reduced by approximnately 50% (Figure 5C), possibly because of the very low basal Cx43 expression in WAT (Figure 2A). Basal glucose uptake, measured as fluorescence intensity in cells after incubation with 2-NBD-glucose, was higher in adipocytes derived from cKO^Tw2^ relative to WT mice. Such difference was significant after a 60-minute incubation, and at 30 minutes in the presence of insulin (Figure 5D). While insulin significantly stimulated glucose uptake in WT adipocytes at 60 minutes, it had no effect in cKO^Tw2^ adipocytes, in which basal glucose uptake at 60 minutes was similar to glucose uptake by WT adipocytes in the presence of insulin (Figure 5D). These results suggest that basal glucose transport may be maximal in cKO^Tw2^ white adipocytes after HFD feeding. There were no differences in expression of lipolysis-related genes, lipoprotein lipase (*Lpl*) and adipose triglyceride lipase (*Pnpla2*), in the iWAT of mutant and WT mice (Figure 5E). Likewise, glycerol release from iWAT cells, an index of lipolysis, was not different between genotypes either in basal conditions or under β-adrenergic stimulation with isoproterenol (Figure 5F).

Because the *Dermo1/Twist2* promoter targets early mesenchymal precursors, we generated cultures of mesenchymal cells from the ear, which represent adipocyte precursors (18). Consistent with the findings from WAT, adipocytes from cKO^Tw2^ ear-derived mesenchymal stem cells (EMSCs) showed increased expression of *Pparg* and *Adipoq*, as well as *Lep* and the insulin-sensitive glucose transporter *Glut4* (Supplemental Figure 5, A and B). Such changes were more evident after 5 days in culture, when adipogenic differentiation is more advanced. By contrast, *Cebpa* and *Glut1* mRNA expression did not differ between control and mutant cells (Supplemental Figure 5, A and B). Confirming effective *Gja1* targeting in these cells (Supplemental Figure 5C), *Gja1* mRNA was barely detectable in adipocytes from cKO^Tw2^ EMSCs. Overall, these results indicate that lack of Cx43 favors adipogenic differentiation, glucose uptake, and adiponectin production without altering lipolysis.

### Gja1 ablation in mesenchymal lineage cells increases locomotor activity, food consumption, and energy expenditure.

**Figure 6:**
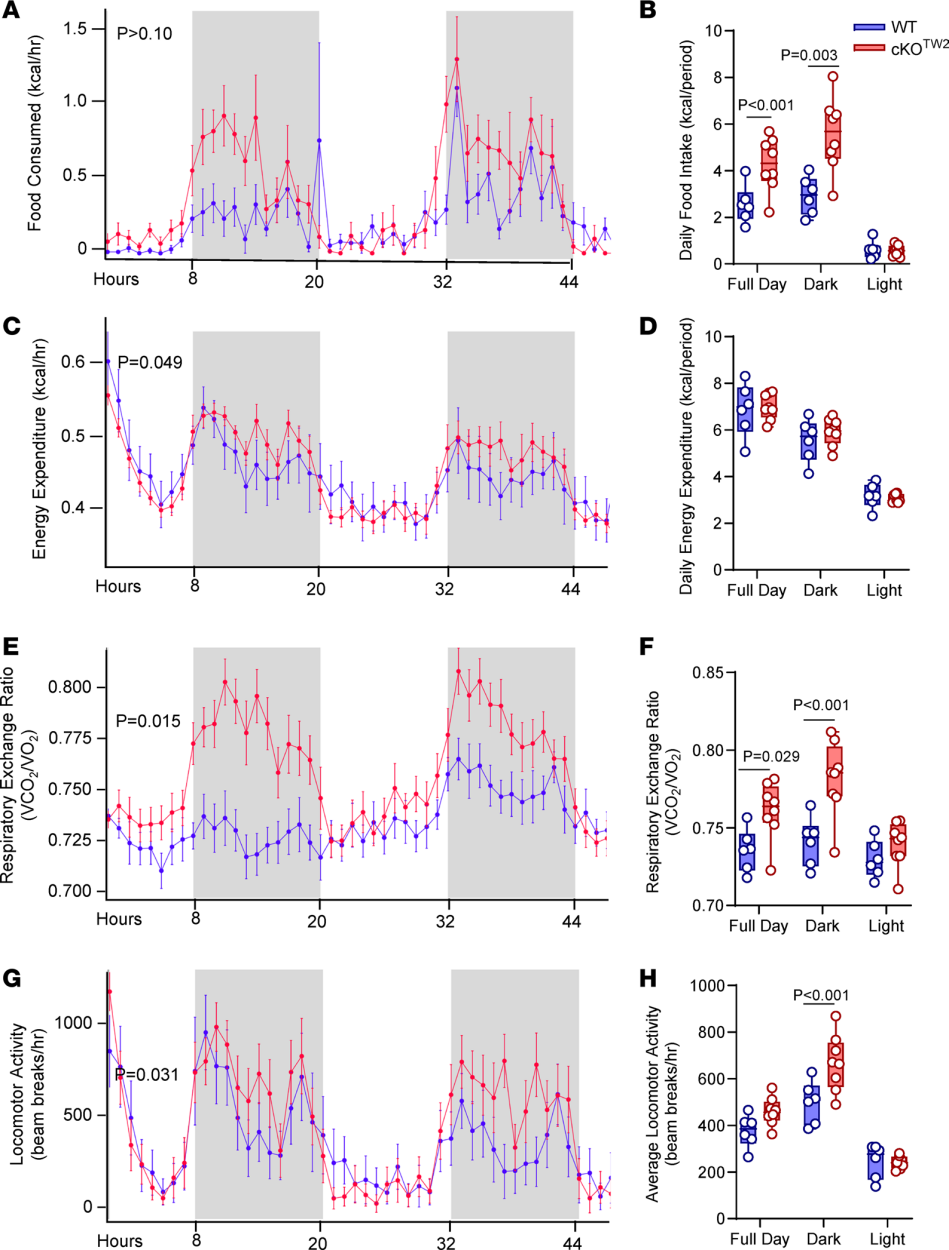
*Gja1* ablation in mesenchymal lineage cells increases locomotor activity, food consumption, and energy expenditure. Five-month-old cKO^TW2^ (red, *n* = 6) and WT (blue; *n* = 8) male mice were placed in metabolic cages after being fed an HFD for 12 weeks, and continuously monitored for 48 hours. (**A** and **B**) Food consumption, (**C** and **D**) energy expenditures, (**E** and **F**) respiratory exchange rate (VCO_2_/O_2_), and (**G** and **H**) locomotor activity. Data are presented as hourly averages (**A**, **C**, **E**, and **G**) and were analyzed using general linear models or ANOVA (detailed results in Supplemental Table 2; *P* values are given for genotype effect), and daily averages over the 2-day experiment for each time period (**B**, **D**, **F**, and **H**), with groups compared using 1-way ANOVA.

Indirect calorimetry was performed on male mice fed an HFD for 12 weeks. Despite less accumulation of body fat and lower body weight (Figure 3, A and B), cKO^Tw2^ male mice consumed more food compared with WT mice, particularly during periods of activity. The genotype difference was not significant when hourly average consumption was analyzed (Figure 6A), but it was evident in the average daily food consumed (Figure 6B). On the other hand, there was a significant genotype effect in hourly energy expenditure, which was higher in cKO^Tw2^ mice during dark cycles (Figure 6C), although such a difference did not emerge in average daily energy expenditure (Figure 6D). Since there was a significant interaction between genotype and body weight in energy expenditure (Supplemental Table 2), these results are consistent with the notion that cKO^Tw2^ male mice consumed more energy per mass unit than their WT littermates, thus offsetting the lower energy consumption expected for their smaller weight. Furthermore, cKO^Tw2^ mice fed an HFD showed higher respiratory exchange ratio relative to WT mice, in both light and dark cycles (Figure 6, E and F, and Supplemental Table 2). They were also more active during the dark cycle (Figure 6, G and H, and Supplemental Table 2), as noted earlier for mice on standard chow diet (Supplemental Figure 3, G and H). These findings indicate that cKO^Tw2^ mice are physically and metabolically more active than WT mice, and under an obesogenic diet they expend more energy and burn more carbohydrates, particularly during physically active cycles.

### Gja1 ablation in mesenchymal lineage cells protects against obesity-induced BAT whitening and increases cold-induced thermogenesis and lipolysis in mice fed an HFD.

**Figure 7:**
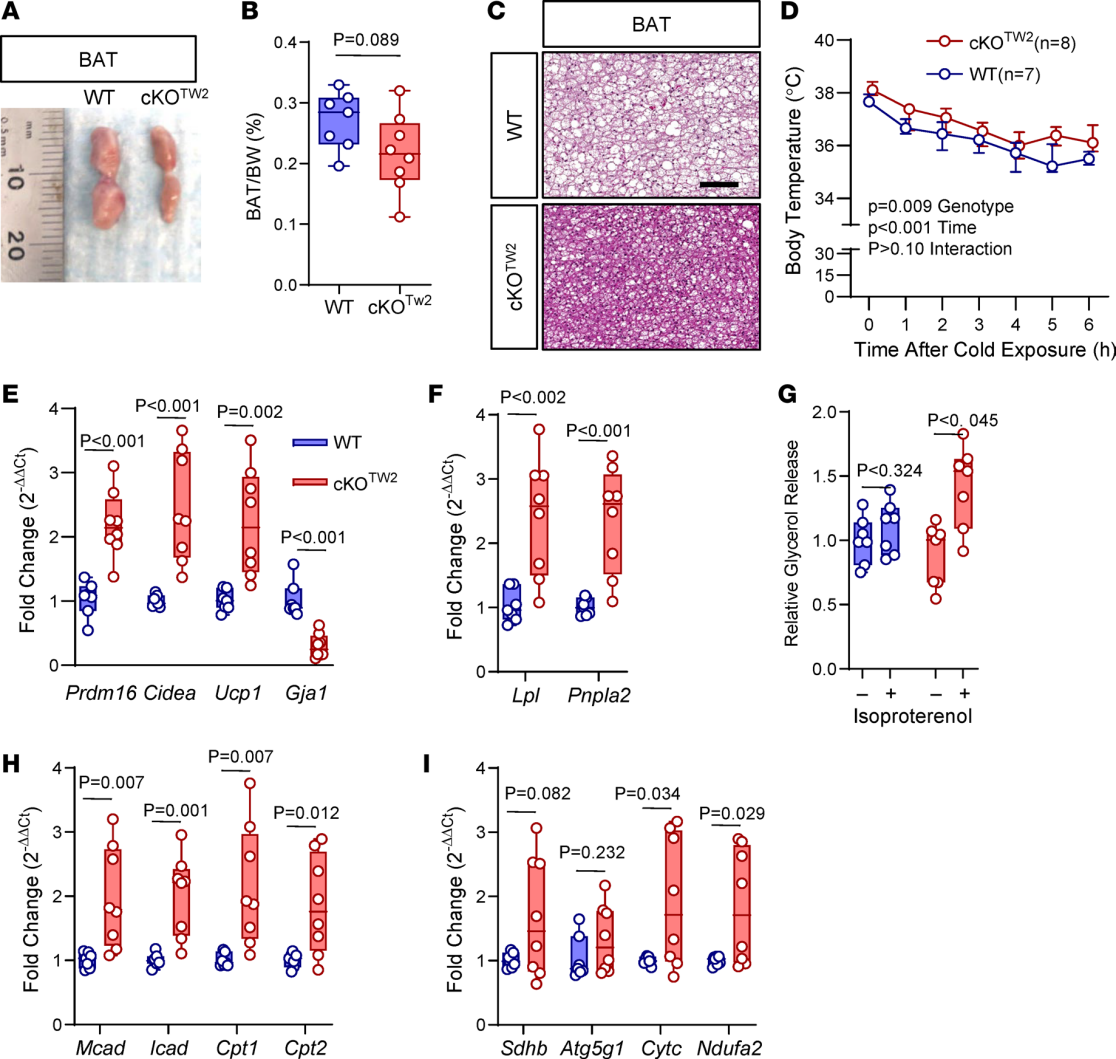
*Gja1* ablation in mesenchymal lineage cells protects from obesity-induced BAT whitening, and increases thermogenesis, lipolysis, fatty acid oxidation, and oxidative phosphorylation in diet-induced obese mice. (**A**) Morphology of suprascapular BAT depots, (**B**) percentage BAT weight relative to body weight, and (**C**) H&E-stained histological sections of BAT of 5-month-old WT and cKO^TW2^ mice fed an HFD for 12 weeks. Scale bar: 100 μm. Each image is representative of 7 WT and 8 cKO^TW2^ mice. (**D**) Core body temperature of WT or cKO^TW2^ mice during exposure to cold temperature (4°C) for 6 hours, after 12 weeks on an HFD. Data are shown as average ± 95% CI; *P* values represent the effect of genotype, time, and their interaction by mixed-effects analysis (genotype, *F* = 9.371, *P* = 0.009; time: *F* = 38.74, *P* < 0.001; time × genotype: *F* = 0.6624, *P* = 0.680). Expression of (**E**) BAT genes and *Gja1* mRNA, and (**F**) lipolysis-associated genes in suprascapular BAT depots of 2-month-old WT (blue) and cKO^TW2^ mice (red) after 12 weeks on HFD. (**G**) Glycerol release relative to tissue weight, in the presence or absence of 10 μM isoproterenol. (**H**) Expression of β-oxidation and (**I**) oxidative phosphorylation genes in BAT from WT and cKO^TW2^ mice after HFD. Box-and-whisker plots represent the interquartile range (box bounds) with median (inside bar); whiskers represent maximum and minimum values. Groups were compared using 2-sided Mann-Whitney *U* test.

BAT increases energy metabolism via fatty acid β-oxidation and regulates body temperature through nonshivering thermogenesis (19, 20). Although BAT mass was significantly lower after HFD in cKO^Tw2^ than in WT mice (Figure 7, A and B), histological sections showed fat accumulation in the BAT of WT mice, whereas in cKO^Tw2^ it appeared only minimally infiltrated with white fat cells after HFD (Figure 7C). Therefore, the larger BAT mass in WT mice reflects BAT whitening under high dietary fat intake, and this change is partially prevented by lack of Cx43. Since Cx43 in BAT is involved in thermoregulation (11), we then studied the response to cold exposure in our mouse model. Both cKO^Tw2^ and WT mice fed an HFD maintained normal body temperature in standard living conditions; however, when exposed to cold (4°C), cKO^Tw2^ mice adjusted to a slightly higher body temperature (approximately 0.5°C) than WT mice (Figure 7D). Reflecting higher thermogenesis, we detected higher levels of mRNA for *Ucp-1* and for the BAT-specific genes *Prdm-16* and *Cidea* in BAT of cold-exposed cKO^Tw2^ mice relative to WT mice after HFD (Figure 7E). As expected, *Gja1* was reduced by 70% in cKO^Tw2^ BAT (Figure 7E), where non-adipocytic cells expressing Cx43 are present. Consistent with increased BAT activity (19), expression of the lipolysis-related genes *Lpl* and *Pnpla2* was significantly higher in BAT of cKO^Tw2^ mice relative to WT BAT after HFD (Figure 7F). While ex vivo lipolysis, measured by glycerol release, in resting conditions was not altered in mutant mice, it significantly increased upon β-adrenergic stimulation with isoproterenol in BAT of cKO^Tw2^ mice, but not in WT BAT (Figure 7G). Finally, BAT of HFD-fed cKO^Tw2^ mice expressed higher mRNA levels of genes involved in fatty acid oxidation and the OXPHOS system (Figure 7, H and I).

### Gja1 ablation in adipocytes does not protect against diet-induced obesity and actually worsens glucose tolerance.

**Figure 8:**
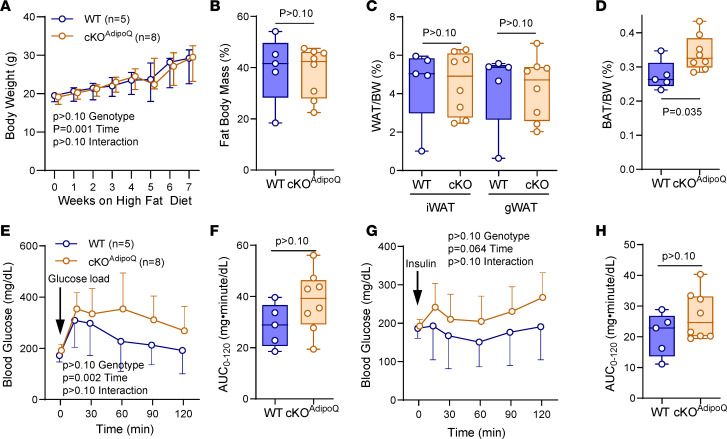
*Gja1* ablation in adipocytic cells does not affect diet-induced obesity and worsens glucose tolerance in mice. (**A**) Body weight of 5-month-old WT (blue) and cKO^Adipoq^ male mice (orange) during 8 weeks on HFD feeding. Data are shown as median ± interquartile range; *P* values represent the effect of genotype, time, and their interaction by mixed-effects analysis (genotype, *F* = 0.1452, *P* = 0.710; time: *F* = 51.53, *P* = 0.001; time × genotype: *F* = 0.3383, *P* = 0.9337). (**B**) Percentage body fat by DXA after HFD in the 2 genotype groups. (**C**) Percentage of inguinal and gonadal WAT (iWAT and gWAT) and (**D**) BAT depots in the 2 genotypes after HFD. (**E** and **F**) Intraperitoneal glucose tolerance test: blood glucose before and after an intraperitoneal load of 1.5 g/kg glucose. Data points represent the mean ± 95% CI; *P* values represent the effect of genotype, time, and their interaction by 2-way ANOVA (genotype, *F* = 2.603, *P* = 0.135; time: *F* = 6.922, *P* = 0.023; time × genotype: *F* = 0.9072, *P* = 0.483). (**G** and **H**) Intraperitoneal insulin tolerance test: blood glucose before and after an intraperitoneal injection of 0.75 U/kg insulin. Data points represent the mean ± 95% CI; *P* values represent the effect of genotype, time, and their interaction by 2-way ANOVA (genotype, *F* = 2.137, *P* = 0.172; time: *F* = 2.822, *P* = 0.064; time × genotype: *F* = 1.014, *P* = 0.418).

Finally, we asked whether more restricted deletion of *Gja1* in adipocytic cells could reproduce some, if not all, changes in energy metabolism observed in cKO^Tw2^. To this end, we generated *Adipoq-Cre;Gja1^fl/fl^* (cKO^Adipoq^) mice. As previously described by other groups (11, 12), adipocyte-restricted deletion of Cx43 did not protect from the effects of HFD. Unlike cKO^Tw2^ mutants, both male and female cKO^Adipoq^ mice gained as much body weight and fat mass as WT mice after 12 weeks on HFD (Figure 8, A and B, and Supplemental Figure 6, A and B). No differences between controls and mutants were noted in WAT depots (Figure 8C and Supplemental Figure 6, D and E). However, BAT mass was higher in cKO^Adipoq^ animals at the end of the HFD period (Figure 8D and Supplemental Figure 6C). In both sexes, mutant and control mice were equally hyperglycemic after HFD, although cKO^Adipoq^ had more severe glucose intolerance relative to WT mice (Figure 8, E and F, and Supplemental Figure 6F). Both mutant and WT males showed no changes in blood glucose after insulin (Figure 8, G and H), whereas females were equally responsive (Supplemental Figure 6H). Thus, adipocyte-specific *Gja1* ablation does not phenocopy the partial resistance to an obesogenic diet observed with broader *Gja1* ablation in mesenchymal precursors, and it actually worsens glucose tolerance, as previously shown (12).

## Discussion

We provide evidence that under obesogenic dietary conditions lack of Cx43 in mesenchymal lineage cells leads to increased physical activity, energy expenditure, and glucose utilization, reduces WAT fat storage, and mitigates the development of the metabolic syndrome induced by high calorie intake in mice. Therefore, the overall action of Cx43 is to restrain energy consumption and store energy in WAT, and it occurs in both sexes, even though the metabolic advantage of Cx43 ablation is more evident in males than in females. This effect is distinct from Cx43 action in mitochondria, where Cx43 favors energy production and mitochondrial biogenesis (12, 21–23). Thus, Cx43 has lineage- and stage-specific actions that can lead to opposite effects on body adiposity and energy metabolism.

Adipose tissue expansion can occur by either increasing the number of adipocytes (hyperplasia) or increasing the size of existing adipocytes (hypertrophy). Adipocyte number in fat depots is determined early in life and remains rather constant in adult life (24, 25). Accordingly, smaller adipocyte size and lower weight of WAT depots in cKO^Tw2^ mice likely reflects reduced fat hypertrophy in the absence of Cx43 under obesogenic dietary stress. However, we cannot exclude a potential contribution of fat hyperplasia, as HFD upregulated adipogenic genes in *Gja1*-ablated WAT and EMSC cultures, and recent evidence suggests that under high caloric stress, adipocyte number can expand around the vasculature of adipose tissues (3, 26). Importantly, smaller WAT adipocytes, increased adipogenic gene expression, reduced BAT whitening, and absence of hepatic steatosis underscores a more metabolically healthy obesity state in cKO^Tw2^ mice (3, 27). Therefore, *Gja1* ablation in *Dermo1/Twist2*-targeted cells reprograms the adipocyte lineage in ways that ultimately enhance the metabolic response to high caloric intake.

Considering its rather broad expression, it is likely that Cx43 acts in multiple mesenchymal-derived cells to drive adipocyte metabolic reprogramming. The enhanced glucose uptake by *Gja1*-ablated WAT and EMSCs and increased adipokine expression by EMSCs we report here point to a cell autonomous action of Cx43 in adipogenic lineage cells. However, *Gja1* deletion in differentiated adipocytes worsens glucose tolerance without major effects on adiposity under high-calorie stress, and *Adipoq*-driven *Gja1* overexpression increases β-adrenergic–stimulated metabolic activity and insulin sensitivity in mice (28). Therefore, adipogenic precursors or cells at early stages of adipogenic differentiation may be key players in the observed phenotype. However, it is likely that other cells targeted by *Dermo1/Twist2* contribute. In an earlier study, we observed modestly lower body weight in mice with *Col1a1*-driven *Gja1* deletion (29), although changes in body mass or composition have not been reported in other models of osteoblast-restricted *Gja1* ablation (14, 30, 31). *Dermo1/Twist2* also targets muscle (32) and to a lesser extent the liver (this study), 2 tissues where Cx43 is also expressed, and increased glucose utilization in these tissues may partly explain the observed phenotype. Partial protection from HFD-induced glucose intolerance and insulin resistance, exactly as we report here, and reduced diet-induced inflammation in gonadal adipose tissue has been observed in mice with selective *Gja1* ablation in macrophages (33). As *Dermo1/Twist2* is expressed by macrophages (34), Cx43 in adipose tissue macrophages may contribute to the phenotype of cKO^Tw2^ mice.

We find that Cx43 is abundantly expressed in adipocyte precursors and rapidly decreases with adipocyte differentiation, as others had also reported (35, 36), while an obesogenic diet upregulates Cx43 expression in BAT. Thus, the function and regulatory mechanisms of Cx43 in the adipose tissue appear to be stage and tissue specific. For example, in mature WAT, sympathetic signals upregulate Cx43 to increase body energy metabolism (11), and in mature BAT, Cx43 is required for mitochondrial integrity and increased metabolic activity (12). However, we find that under obesogenic stress, cKO^Tw2^ mice generate more heat after cold exposure, and the BAT of cKO^Tw2^ mice is more metabolically active than the BAT of control mice. Therefore, while the increased physical activity may explain, at least in part, the higher body temperature upon cold exposure, we hypothesize that during embryonic and early postnatal development, lack of Cx43 reprograms BAT precursors toward a more metabolically active phenotype, thus overriding the consequences of a lack of Cx43 on mitochondrial function. Our data also suggest that Cx43 in WAT favors fat accumulation and reduces energy expenditure, particularly in obesogenic conditions. The smaller size of fat depots in cKO^Tw2^ mice even under standard dietary regimens may imply that a lower number of adipocytes develop during the early postnatal period, and this may contribute to reduced fat accumulation in obesogenic conditions.

Specific functional domains of Cx43 may have different biologic roles in adipocytes; for example, while chemical inhibition of gap junctional intercellular communication or *Gja1* silencing by siRNA in adipocyte precursors inhibits adipogenic differentiation (7, 35, 37) and prevents WAT beiging in response to cold (11), intercellular communication is dispensable for repression of autophagy by Cx43 (38). Furthermore, Cx43 interacts with the mitochondrial machinery (12) and binds to respiratory complex I (22), actions that do not necessarily require gap junction channel formation. Thus, channel function may be necessary for Cx43 modulation of adipogenesis and the metabolic response to an obesogenic diet, while Cx43’s effects on cellular glucose metabolism and energy production may be linked to channel-independent functions. We also find that, similar to the osteogenic lineage, adipocytes express Cx45, which like Cx43 is downregulated during adipogenic differentiation. These 2 connexins form gap junctions of different biophysical properties (39), but it is possible that Cx45 may in part compensate for lack of Cx43 in establishing intercellular communication among adipocytes.

The increased locomotor activity certainly contributes to the increased energy expenditure in cKO^Tw2^ mice, particularly under an obesogenic diet, the result of increased fuel consumption by muscles and cold exposure while ambulating (40). Notably, while on a standard diet cKO^Tw2^ mice use more energy from fat, under obesogenic stress they burn more carbohydrates and utilize more glucose than control mice, particularly during active nocturnal cycles. Such excess energy consumption likely contributes to the lower fat accumulation and better glucose tolerance in cKO^Tw2^ mice on HFD. The reasons for the higher physical activity of mutant mice remain unclear. It is unlikely that this abnormality has a central cause, because the *Dermo1/Twist2* promoter does not target the nervous system, even though Cx43 is present in glial cells (41). Upregulation of *Adipoq* mRNA in WAT and in differentiating EMSCs supports the idea that increased production of adiponectin or other adipokines, or endocrine factors may in part explain increased glucose utilization and energy consumption by cKO^Tw2^ mice. We and others have shown that Cx43 modulates the expression of factors relevant to bone homeostasis via transcriptional regulation (42, 43), and a similar mechanism might be at play in the adipogenic lineage. It is also possible that adipokines produced by BAT (batokines) may play a role, as Cx43 is abundantly expressed in BAT and is upregulated by high caloric intake.

**Figure 9:**
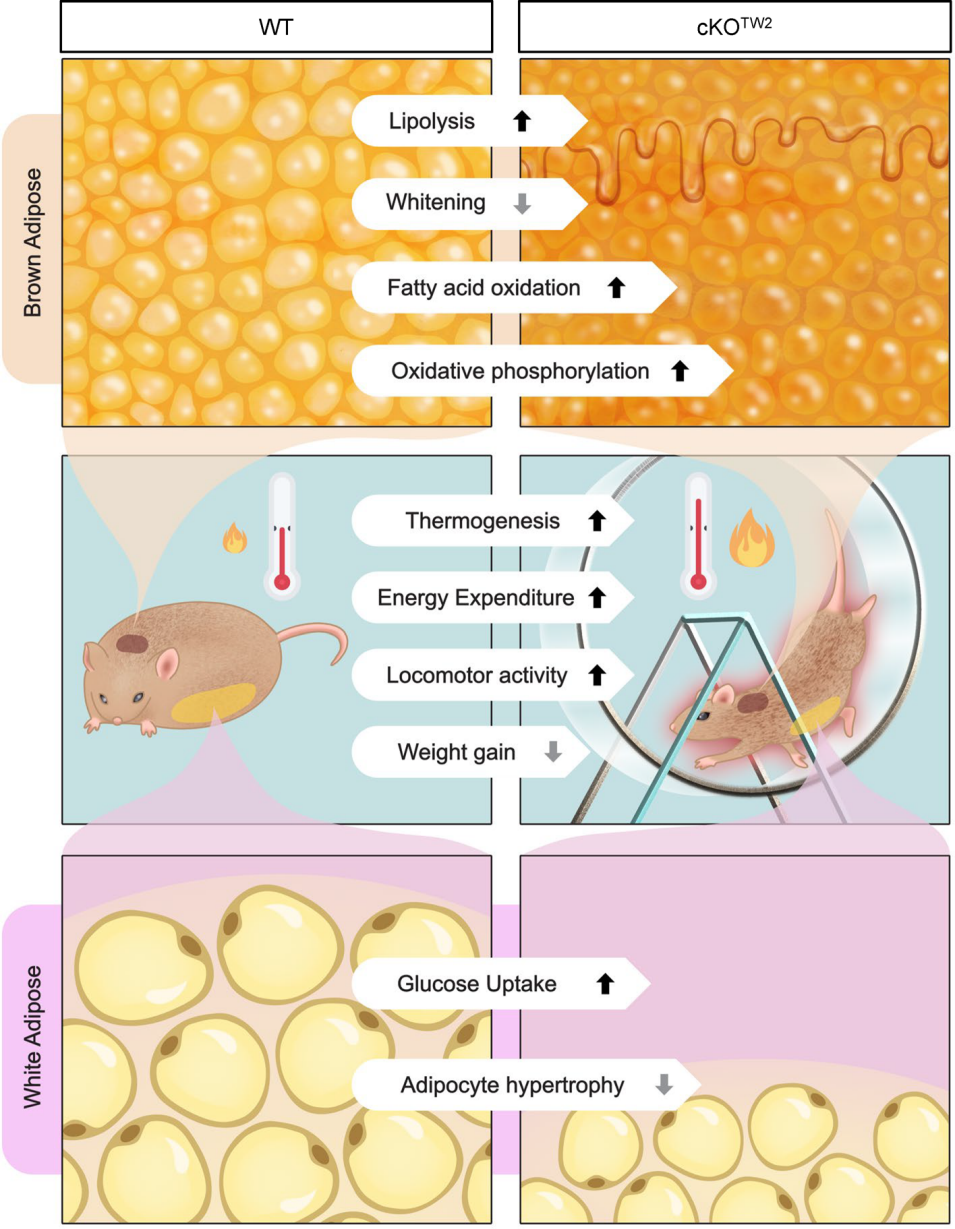
Schematic representation of the effect of *Gja1* ablation on the metabolic response to an HFD. Left column: In normal mice, high dietary calorie intake changes energy metabolism, resulting in excess energy storage in fat depots and other organs, leading to obesity, hyperinsulinemia, high serum lipids, and glucose intolerance. In WAT depots (bottom row), fat accumulation occurs primarily by adipocyte hypertrophy; in BAT (top row), it leads to whitening as cells become engulfed by lipid droplets. Right column: Genetic ablation of *Gja1* in the mesenchymal lineage (cKO^Tw2^) mitigates these effects of high calorie intake, resulting in reduced BAT whitening and higher BAT activity (increased lipolysis, fatty acid oxidation, and oxidative phosphorylation), smaller WAT depots, and increased glucose uptake and utilization. At the organism level (middle row), Cx43-deficient mice are more active and more cold tolerant, burn more energy, and utilize more glucose than control littermates under high calorie intake. We propose that the increased energy consumption for physical activity and thermogenesis reduces fat accumulation, WAT hypertrophy, and BAT whitening, resulting in less severe obesity, partially preserved glucose tolerance, and better circulating lipid profile than in normal mice.

We propose the following biologic mechanism (Figure 9): absence of Cx43 in mesenchymal lineages results in reduced volume of WAT depots and reduced WAT hypertrophy and increased glucose utilization by WAT under high caloric intake. In parallel, there is increased fuel utilization, lipolysis, and thermogenesis in BAT, and reduced BAT whitening. These changes result in increased energy expenditures, better glucose tolerance, and reduced weight gain and overall better metabolic response to obesogenic stress. Whether increased locomotor activity of Cx43-deficient mice is the main driver of, or is secondary to, a better metabolic balance remains to be determined. We propose that the less severe metabolic syndrome developing in *Gja1*-ablated mice overrides a poorer glucose tolerance caused by loss of Cx43 in the mitochondria of mature adipocytes, thus limiting the impact of high-calorie intake on energy metabolism (Figure 9).

The increased glucose uptake by cKO^Tw2^ WAT and EMSCs suggest that adipose-derived precursors in adult cKO^Tw2^ mice might retain a higher metabolic activity trait in adult animals, and this could be leveraged for therapeutic potential. Infusion of adipose-derived stem cells from human WAT or visceral adipose tissue into obese mice improves glucose metabolism and lipid profiles and reduces body weight gain relative to control mice (44, 45), an effect mediated by secretion of adipokines, antiinflammatory cytokines, or angiogenic factors (46). Regardless of the mechanism, if this cell-based therapy can be translated to humans, then it is conceivable that genetic or pharmacologic interference with Cx43 function in adipose-derived stem cells before infusion in obese individuals may enhance their metabolic activity. This hitherto unknown aspect of Cx43 biology offers a promising new therapeutic target for improving metabolic balance in diabetes and obesity.

## Methods

### Sex as a biological variable.

Fundamental experiments on the effects of HFD and regular diet were performed and presented separately for male and female mice. Since similar results were obtained in both sexes, with some relatively minor differences in insulin sensitivity, more detailed studies on energy metabolism and adipose tissue biology were performed only in male mice, which exhibited a more severe metabolic phenotype, as detailed under Results.

### Mouse models.

The *Dermo1/Twist2-Cre* and *Gja1^fl/fl^* mouse lines and the mating strategy to obtain cKO^Tw2^ mice have been previously described (13). Adipoq-Cre mice were obtained from The Jackson Laboratory (strain 028020). All mice were housed in a standard temperature- and humidity-controlled environment with a 12-hour light/12-hour dark cycle. A diet-induced-obesity mouse model was established by feeding an HFD containing 60% of calories from fat (Research Diets, D12492) for 8 or 12 weeks. After the iGTT and iITT (see below), mice underwent dual energy x-ray absorptiometry for body fat composition analysis, followed by fat dissection and collection of BAT from suprascapular depots, iWAT, and gWAT. For cold exposure experiments, mice fed an HFD for 8 weeks were transferred to prechilled cages containing prechilled water and food in a 4°C cold room. Core body temperature was measured hourly by rectal probe (RET-3, ThermoWorks).

### Blood biochemistry.

For the iGTT, mice fasted for 6 hours were administered a bolus of D-glucose (1.5 g/kg) by i.p. injection. For iITT, mice fasted for 6 hours were administered insulin (0.5 U/kg for chow diet–fed mice and 0.75 U/kg for HFD-fed mice). Blood glucose levels in the tail vein were monitored at various time points (0, 15, 30, 60, 90, and 120 minutes) using the Glucocard Vital blood glucose meter (Arkray, Inc.). Serum lipids and hormones were determined by the Washington University Diabetes Models Phenotyping Core, Diabetes Research Center.

### Indirect calorimetry.

Respiratory gas measurements, food consumption, and activity were investigated using the PhenoMaster (TSE Systems), also available in the Washington University Diabetes Research Center. Mice were placed in the metabolic cages and monitored for 48 hours. Metabolic variables were elaborated using CalR (https://calrapp.org/). The tool was set to analyze the interaction between body weight (mass effect) and mouse genotype and applies general linear models when a significant interaction exists, or ANCOVA when there is no interaction. ANOVA was used for mass-independent variables (47). Data used in this study are provided in the supplemental material (Supplemental Tables 4 and 5).

### RT-qPCR.

Total RNA was isolated from fat tissue extracts or EMSCs using an RNeasy Mini Kit (Qiagen). Reverse transcription was performed using 500 ng of total RNA and iScript reverse transcription super mix (Bio-Rad, 1708891). PCR reactions were performed in a 96-well format on an ABI QuantStudio 3 using Fast SYBR Green Master Mix (ABI, 4385612). β2-Macroglobulin was used for normalization, and relative expression was calculated by the 2^−ΔΔCT^ method. Primer sequences are listed in Supplemental Table 3.

### Histology.

Tissues, fat, and liver were dissected out, fixed in 10% neutral buffed formalin overnight at room temperature, and then were processed for paraffin embedding. Sections (5 μm thickness) were stained with H&E as previously described (48).

### Immunohistochemistry.

Formalin-fixed, paraffin-embedded adipose tissue sections were deparaffinized with xylene, rehydrated, and treated with 0.3% hydrogen peroxide in methanol for 15 minutes to suppress the endogenous peroxidase activity. Antigen retrieval was achieved by placing the samples in a pressure cooker and incubating for 3 minutes at full pressure in citrate buffer (10 mM citric acid, pH 6.0), followed by gradual cooling to room temperature. Sections were then blocked using serum blocking solution (Invitrogen Histostain-SP Kit) and incubated overnight with primary antibody against mouse Cx43 (unconjugated F-7, mouse, Santa Cruz Biotechnology) diluted 1:200 in PBS/0.1% Triton X-100 (Sigma-Aldrich) at 4°C. The next day, sections were washed 3 times with PBS and incubated at room temperature with biotinylated universal secondary antibody (Life Technologies). After washing with PBS 3 times, secondary antibodies were visualized using a Vectastain ABC kit (Vector Laboratories, PK-4000) and ImmPACT DAB Substrate Kit, Peroxidase (HRP) (Vector Laboratories, SK-4105). Sections were then counterstained using Gill II Hematoxylin followed by washing in PBS and dehydration in ascending ethanol series and xylene. Sections were imaged using a Hamamatsu NanoZoomer 2.0-HT system.

### Western blotting.

As previously described (49, 50), total protein was extracted using RIPA buffer (Cell Signaling Technology, 9806) containing protease inhibitor (Thermo Fisher Scientific, A32963) and phosphatase inhibitor (Thermo Fisher Scientific, A32957). Proteins (15 μg) were separated in SDS-PAGE gels by electrophoresis and transferred onto PVDF membranes (Millipore). Membranes were blocked with 5% nonfat dry milk (Cell Signaling Technology, 9999) in PBS-T (Thermo Fisher Scientific, 28352), and blotted using antibodies against Cx43 (Sigma-Aldrich, C6219), Cx45 (Santa Cruz Biotechnology, sc-374354), Cx40 (Santa Cruz Biotechnology, sc-365107), or β-actin (Cell Signaling Technology, 4970). Immune reactions were detected using an HRP-conjugated anti-rabbit secondary antibody (Cell Signaling Technology, 7074).

### Lipolysis assay.

Dissected intrascapular BAT and inguinal adipose tissues were incubated in high-glucose DMEM containing 2% fatty acid–free BSA (Sigma-Aldrich, A8806) for 30 minutes at 37°C. To analyze basal lipolysis, tissues were transferred into 96-well plates containing 150 μL of high-glucose DMEM supplemented with 2% fatty acid–free BSA and incubated for 1 hour at 37°C. To analyze agonist-stimulated lipolysis, tissues were preincubated in high-glucose DMEM supplemented with 2% fatty acid–free BSA with 10 μM isoproterenol for 30 minutes. Tissues were transferred to 96-well plates containing the same medium and incubated for 1 additional hour. Basal and stimulated lipolysis were determined by measuring glycerol content in the media using free glycerol reagent (Sigma-Aldrich, F6428) and glycerol standard solution (Sigma-Aldrich, G7793). Lipolysis was normalized to protein amount.

### Adipogenic cell culture and differentiation.

For EMSCs, external ears from WT and cKO^TW2^ mice were collected in ice-cold HBSS containing penicillin/streptomycin (Gibco) and primocin (Invivogen). Ears were cut into small pieces in HBSS containing 2 mg/mL collagenase I (Worthington Biochemical Corporation) and digested for 1 hour in a 37°C shaking water bath. Digested ears were filtered through 70-μm cell strainers (BD Biosciences) and pelleted by centrifugation at 327*g* for 10 minutes. Cells were resuspended using EMSC culture media (DMEM/F12 containing 15% FBS and 10 ng/mL FGF) and seeded in 24-well plates at 2 × 10^5^ cells/well and incubated for 2 days. For isolation of SVF preadipocytes, iWAT was isolated from 2-month-old WT mice immediately after sacrifice. The tissue was digested with collagenase type I at 37°C for 30 minutes, and then filtered using a 70-μm cell strainer. After centrifugation, the pellet containing the SVF was collected. The cells were resuspended and cultured in DMEM/F12 media with 10% FBS, and the media were changed every other day. To induce adipogenic differentiation of EMSCs, cultures were switched to an adipogenic medium containing 5 μg/mL insulin, 1 μM dexamethasone, 500 μM IBMX, and 5 μM rosiglitazone (all Sigma-Aldrich). After 2 days, medium was changed to DMED/F12 containing 10% FBS, 5 μg/mL insulin, and 5 μM rosiglitazone for an additional 2 days. Cells were then incubated in 10% FBS–containing DMEM/F12 until lipid accumulation occurred. For EMSCs, cultures were switched to serum-free medium for 2 hours before insulin exposure. The IngWAT mouse immortalized preadipocyte cell line (Millipore, SCC211) was induced to differentiate using AdipoLife DfKt-2 Adipogenesis media (LifeLine Cell Technology), with medium changed every 2 days. To ascertain adipocyte differentiation, some cultures were stained with Oil Red O (ScienCell) following the manufacturer’s instructions.

### Glucose uptake.

Differentiated adipocytes from WT and cKO^TW2^ EMSCs were cultured in growth medium until confluent, and then switched to serum-free media for 1 hour before incubation in 100 μM 2-NBD-glucose (a fluorescent deoxyglucose analog) with or without insulin for 30 or 60 minutes. Fluorescence intensity was measured according to the manufacturer’s instructions (Glucose Uptake Cell-based Assay Kit, Cayman Chemical, 600470).

### Statistics.

Group data are presented as box-and-whisker plots with median and interquartile range; whiskers represent maximum and minimum values. Unless otherwise noted, repeated measures are plotted as mean ± 95% confidence interval (CI). Differences between groups were assessed using the Mann-Whitney *U* test, and repeated measures were analyzed by 2-way analysis of variance (ANOVA) or mixed-effects models, in cases of missing data points; these were followed by Tukey’s test to adjust *P* values for multiple comparisons. Exact *P* values are provided, and a *P* value of less than 0.05 was considered significant. Data were managed in Microsoft Excel, plotted, and analyzed using Prism 10.0 (GraphPad Software).

### Study approval.

All the procedures reported here were approved by the Institutional Animal Care and Use Committee at Washington University (protocol number 20-0029) and followed the Animals in Research: Reporting of In Vivo Experiments (ARRIVE) guidelines.

### Data availability.

All underlying data are included in a the supplemental Supporting Data Values XLS document. Indirect calorimetry source data are provided in Supplemental Tables 4 and 5.

## Author contributions

SYL designed the research studies, conducted experiments, acquired data, analyzed data, and wrote the manuscript. FF designed the research studies, conducted experiments, acquired data, and wrote the manuscript. TS conducted experiments, acquired data, and wrote the manuscript. IPC and GL conducted experiments and acquired data. ACR analyzed data, generated graphs, and wrote the manuscript. RC designed the research studies, analyzed data, wrote the manuscript, and secured funding.

## Supplementary Material

Supplemental data

Unedited blot and gel images

Supplemental table 4

Supplemental table 5

Supporting data values

## Version 1 02/13/2024

In-Press Preview

## Version 2 03/22/2024

Electronic publication
