# Supplementary material for: Connexin43 in mesenchymal lineage cells regulates body adiposity and energy metabolism in mice
Source: JCI Insight. 2024 Feb 13;9(6):e170016. doi: 10.1172/jci.insight.170016 (PMC11063945; doi:10.1172/jci.insight.170016)
Supplement: Unedited blot and gel images [file jciinsight-9-170016-s069.pdf]

Unedited Western Blots for Figure 2B

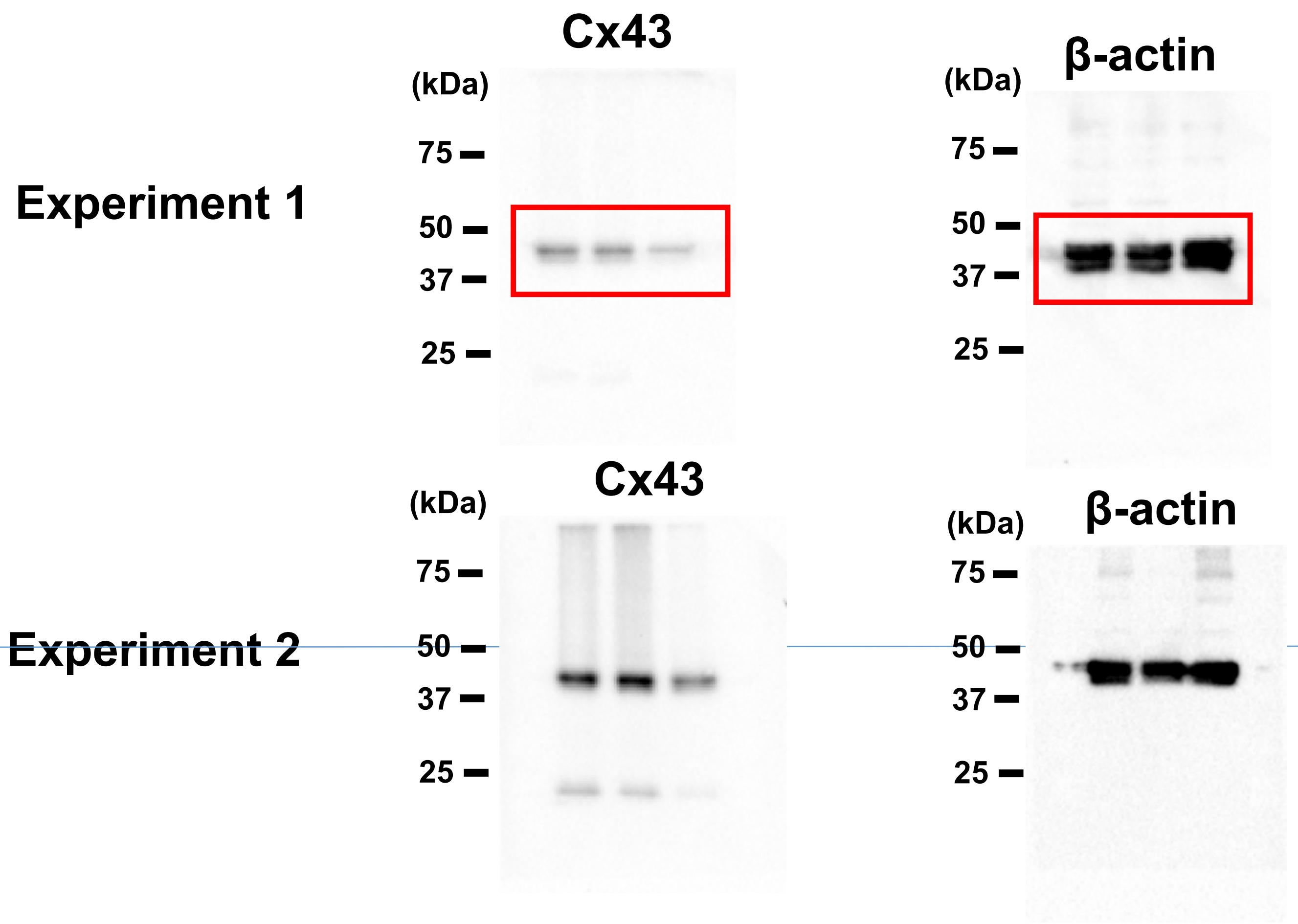

For each experiment, 2 separate gels were loaded with the same whole cell lysates, run, and blotted with the antibody indicated. Red rectangles indicate the areas used for Fig. 2B.

Unedited Western Blots for Figure 2C

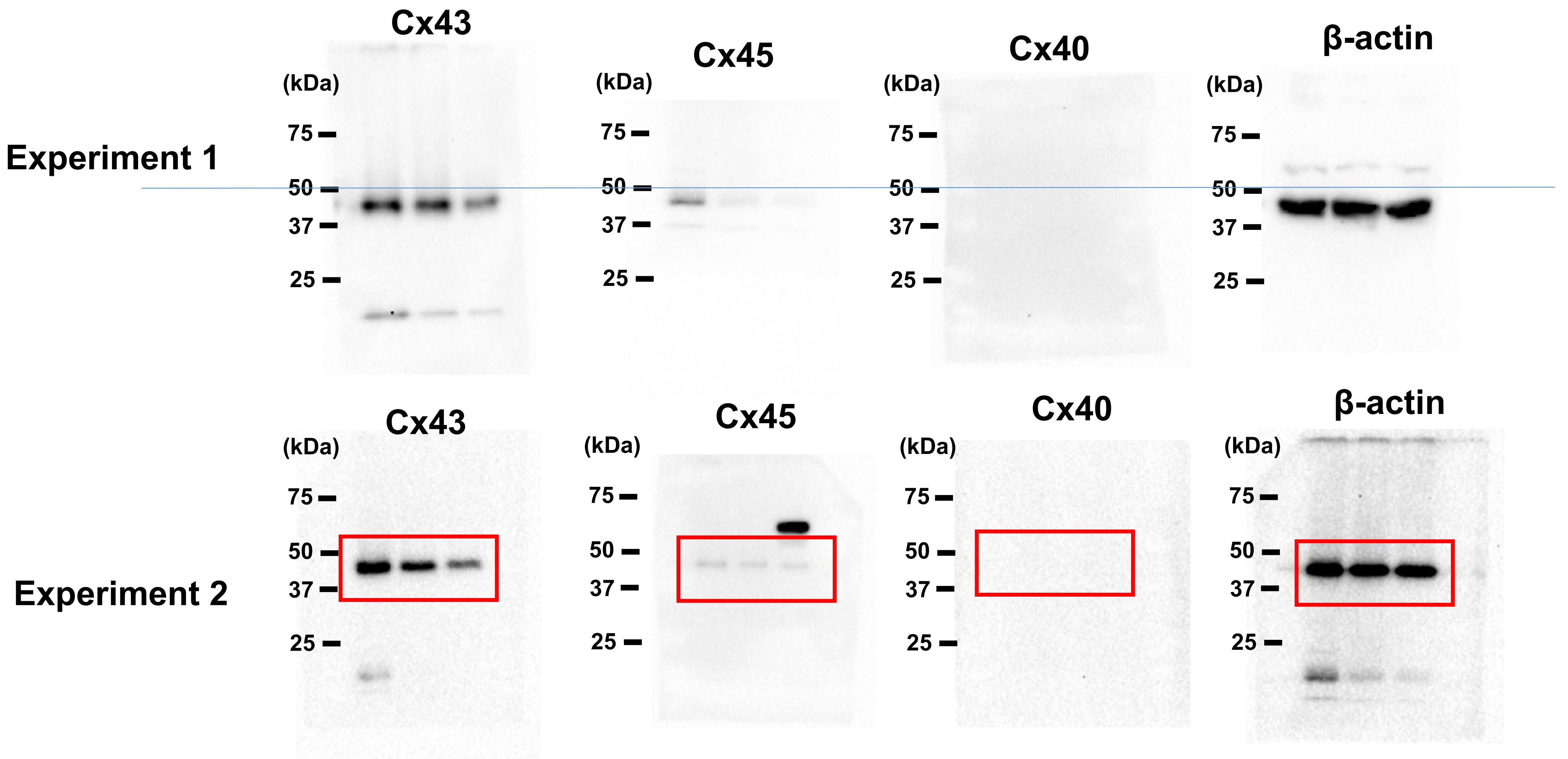

For each experiment, 4 separate gels were loaded with the same whole cell lysates, run, and blotted with the antibody indicated. Red rectangles indicate the areas used for Fig. 2C.
